# The Clinical Society of London

**Published:** 1889-06

**Authors:** 


### CLINICAL SOCIETY OF LONDON.

### FIVE CASES OF DISEASE OF THE MIDDLE EAR COMPLICATED BY SUPPURATION IN ITS VICINITY.

Mr. Arbuthnot Lane read brief abstracts of these cases, which he had had under his care during the last few months, and in which the complications differed in each case. He felt justified in bringing them forward for the reason that, though such cases were not uncommon, the symptoms indicative of the presence of each possible complication, or of the existence of any complication at all, were frequently very indefinite, and often insufficient to enable the surgeon to make anything approaching a certain diagnosis, and the time for decision was very often so short that he could not be too familiar with the varying details of a number of cases. He considered that the risks involved in operative interferences were comparatively slight, and in doubtful cases the middle ear and its vicinity should be thoroughly explored. Case I was that of a woman, aged 38, who on August 21st, 1888, "ricked" her neck. She felt at the time as if something had given way in her right ear, which up to that time had caused her no trouble whatever. At the same time, she noticed that pus was escaping from the meatus. She was then affected with headache, gradually increasing in intensity, in the right half of the head, and with pain beneath the upper half of the sterno-mastoid. Mr. Lane saw her on September 5th. Pus welled out freely from an aperture in the membrana tympani. The right ear was quite deaf. There was no optic neuritis. There was a tender and painful swelling beneath the upper half of the sterno-mastoid. The mastoid process was tender on percussion. The mastoid cells were freely opened up with a gouge, and were found to contain offensive pus, and a channel was cut into the middle ear from which pus and caseous material were removed. The lateral sinus was seen to be healthy. A metal drain was fixed in the middle ear, and that cavity irrigated daily. On August 2nd, an abscess beneath the sterno-mastoid was opened, and a probe could be introduced through it into the mastoid process. The patient was now in excellent health. Case II, a girl, aged 12½, was admitted into Guy's on September 18th, 1888, under the care of Mr. Howse. In August, 1887, an abcess formed in her right ear, and was well in a short time. She had no further trouble till a fortnight before her admission, when she was attacked with great pain and swelling in and about the same ear. On admission there was a copious discharge from the meatus, and a large fluctuating swelling extending for about two inches upwards, backwards, and forwards from the ear. This abscess was freely incised and irrigated, and was found to communicate through the meatus between the cartilage and bone with the middle ear. After this the child lost all pain, and was going on remarkably well till, on September 23rd, she became a little drowsy in the morning. The drowsiness increased during the day, the left pupil became dilated, and the child vomited. At 10.45, Mr. Lane saw the child in Mr. Howse's absence. The respirations were then extremely slow, the child was quite unconscious, and there was no optic neuritis. Considering that an abscess in the temporo-sphenoidal lobe had probably ruptured into the lateral ventricle, he proceeded at once to open it, and removed from the temporo-sphenoidal lobe a large quantity of offensive pus and fluid. A drainage tube was inserted, and the cavity thoroughly irrigated. The respirations gradually resumed their normal frequency, and the child flinched when hurt. The improvement did not last very long, for on the following morning the respirations became again very slow, and the child died. The *post-mortem* examination verified the diagnosis. This abscess had given no indication of its presence till within eighteen hours of death, and then the symptoms which arose were consequent upon an extension of the inflammation. Case III, a girl, aged 12, had always been healthy till September 13th, 1888, when she caught cold; she then observed that there was a purulent discharge from her right ear which ceased in a few days. On September 25th, when blowing her nose, she felt something crack in it, and on the following day pus escaped from the meatus, and she complained of pain in the back of her head. The discharge continued, and the headache increased in intensity till October 4th, when Dr. Heron was called in, and he asked Mr. Lane to see her with him. They found no pain, tenderness, or swelling about the ear or mastoid process. Fœtid pus escaped through a hole in the membrane. There was double optic neuritis and retraction of the head. She answered questions readily. The right ear was quite deaf. She was admitted into Guy's that afternoon. The mastoid process was opened up, and a localised collection of pus found in its deepest part. The lateral sinus was healthy. About a drachm and a half of very offensive pus was removed from between the posterior surface of the petrous bone and the dura mater. This cavity and the middle ear were drained, and irrigated daily afterwards. October 5th, the child had quite lost the headache, and the retraction seemed less marked. There was a considerable escape of pus. She progressed very favorably till October 8th, when the temperature ran up to 105.6° at 2 A. M., and the headache and drowsiness returned; she gradually grew worse, and died on October 14th. The *post-mortem* examination showed that the dura mater pressing the wall of the abscess-cavity had sloughed, and the arachnoid cavity below the tentorium was in communication with the cavity of the abscess, and full of pus. Case IV was admitted under Dr. Perry, August 15th, 1888. Since he was eighteen months old he had suffered from periodical attacks of discharge from the right ear preceded by pain every two months. A polypus was removed in November, 1887. On August 8th, 1888, one of these attacks came on, and on the following day the discharge appeared. He vomited, and was dull and feverish. On August 15th, a small polypus was removed. On his admission on August 15th, he was in a semi-comatose state. Both discs were swollen. A scanty, offensive discharge escaped freely through a hole in the membrana tympani. August 16th, he had a rigor. Mr. Lane was asked by Dr. Perry to see the boy, and he opened up the mastoid cells freely, but found no pus. August 18th, the boy was better in the morning, but became dull in the afternoon. He had a rigor. August 18th, another rigor occurred. Mr. Lane gouged away a further portion of the mastoid process, and opened up a small collection of very offensive pus in it immediately adjacent to the lateral sinus. Similiar material covered the sinus, whose outer wall and contents were soft and fœtid. The lateral sinus was cleared of its septic contents upwards and downwards till apparently healthy clot was reached. The internal jugular vein was tied below the thrombus to prevent any escape of blood from the sinus, and the entrance of antiseptic agents or septic material into the circulation. A drainage-tube was inserted into the lateral sinus, which was irrigated daily. After the operation the temperature rarely exceeded 100°, and the boy recovered very rapidly. He left the hospital on October 3rd, and had since continued in excellent health. Case V, W. S., aged 7¾. Since 3½ years of age he had suffered from attacks of earache and otorrhœa on the right side at intervals of two or three months. On December 17th, 1888, he was attacked by earache, headache, and sickness. On December 23rd, he had two rigors, and vomited three times. On December 24th, he had two rigors in the morning, and was admitted at 12 A. M. under Mr. Lane's care. His temperature was 104°, and he was drowsy. He had severe headache on the right side, and tenderness about the mastoid process; both discs were blurred. Mr. Lane operated at once, and found the mastoid cells tensely distended with fœtid pus, the lateral sinus bathed in pus which had escaped from the mastoid process—it was, however, not thrombosed; pus had also escaped from the mastoid process, and collected beneath the sterno-mastoid. The temporo-sphenoidal lobe and the upper and posterior surfaces of the petrous bone were found healthy. The child lost the headache immediately, but he had two rigors on the following day and one on the day after. He suffered continuously from great pain in the abdomen, and his motions were very loose and offensive. For two days his temperature became more moderate, and except that the pain in the abdomen came on at intervals, he seemed much better; the optic neuritis cleared up rapidly. On the 29th his temperature became very irregular and from that date he became rapidly worse, complaining of pain in the abdomen, and suffering from diarrhœa. On January 4th, the base of the right lung had become dull, but aspiration with a morphine needle only yielded blood. The boy rapidly became worse, and died on January 6th. Multiple abscesses were present in both lungs, and especially in the right one. There was no thrombosis of the lateral sinus, and the brain was apparently normal.—Mr. Godlee asked whether Mr. Lane thought it advisable always to explore the groove of the lateral sinus in all cases of suppuration.—Mr. Lane, in reply, said he had no difficulty in clearing out the lower part of the sinus. He had ligatured the jugular vein as high in the neck as he could get, and he thought it was wise to do this under the circumstances.

### LEONTIASIS OSSEA IN A MAN 24 YEARS OF AGE.

Mr. Bland Sutton communicated the details of this case. The bones of the face were chiefly affected. The symmetrical bony protuberances usually found on each side of the nose in this remarkable disease had been removed at Charing Cross Hospital. As the lower jaw had of late increased greatly in size, its right half was removed. The enlarged right upper jaw had encroached upon the orbit, producing pryotosis, and on the nasal fossa, obstructing respiration. On account of these complications it was successfully removed. The patient recovered rapidly from the operation, but the pressure on the optic nerves still remained, indicating that the sphenoid bone was implicated in the disease. Until the operation was performed on the lower jaw the case was regarded as one of exostosis, but on exposing the jaw its true nature was obvious.—The President said it was the only case he had heard of in which the lower jaw had been operated upon, but he himself had on several occasions operated upon and even removed the upper jaw, though he had come to the conclusion that this was not a desirable thing to do, and in future he would content himself with removing the excrescences of bone.

### CASE OF TOTAL EXTIRPATION OF THE LARYNX FOR EPITHELIOMA, WITH RECOVERY AND A USEFUL VOICE.

Dr. Greville Macdonald and Mr. Charters Symonds read notes of this case. The patient was a man, aged 41, married, a commercial traveler, who was first seen by Dr. Macdonald on April 6th, 1888. He had loss of voice, which had been increasing for the previous six months; he had never had syphilis and his family history was excellent; he was a moderate drinker, but a heavy smoker. He complained of slight dyspnœa on exertion; he had no pain or cough, neither expectoration nor hæmoptysis. The laryngoscope revealed a large, irregularly lobulated, greyish-pink neoplasm, occupying the anterior three-fourths of the rima glottidis; much of the left cord was completely concealed by it, as well as the anterior half of the right cord. On inspiration it fell, leaving the right cord free. The posterior surface of the growth presented a small superficial ulceration. Examination with the probe indicated an extensive attachment to the infraglottic portion of the right cord, while the ventricular band was quite free from implication. Both cords moved freely, except so far as they were impeded mechanically. The posterior fourth of the left cord—the only portion visible—was greatly thickened and much congested. There were no enlarged glands. Dr. Macdonald then expressed the probable need of an external operation. A small portion of the growth was removed and examined and seemed to be an epithelioma. On April 14th he urged extirpation of half the larynx, but an adverse consultation and the patient's wishes being against this, Dr. Macdonald removed the whole of the projecting neoplasm with the forceps on April 24th; hæmorrhage was rather profuse. The growth was soft and friable. On the following day the voice was completely restored; there was no trace of growth, but the left cord was uniformly thickened, rounded, and deeply congested, its movements being perfect. A week later there was seen at the junction of the anterior with the middle third, on the upper surface, a sharply-defined silver surface, flush with the mucous membrane, two or three lines in diameter. On examining this surface with the probe, it proved to be minutely villous. This appearance remained unchanged till July 30th, when the villous surface appeared slightly raised above the surrounding surface, which was yellowish and opaque. On August 26th there was scarcely any change. On September 8th the voice was worse, the growth had taken a fresh start and was projecting over the margin of the cord, and extending to the anterior commissure. Again an operation was urged, but another opinion, while admitting malignancy, proved adverse to operation. On October 20th the growth had advanced rapidly, appearing very like its first aspect in April, though more congested. Again an external operation was urged. Mr. Charters Symonds was consulted, and asked to perform the operation. —Mr. Symonds then described the operation, which was performed October 28th, 1888. The trachea was opened above the isthmus, and Hahn's tube inserted, after which chloroform was administered. Then a median incision was carried from the upper end of the incision to the hyoid bone. The hyoid, like the thyroid, had to be dissected away. Then, the thyroid cartilage being fully exposed, it was divided in the middle line, and, being ossified, gave some trouble. The crico-thyroid and thyro-hyoid membranes were divided, with the crico-thyroid muscle, when the ala could be drawn aside and a view obtained. The growth was found to be longer than expected, and to involve the whole of the left cord, while the anterior part of the right looked granular and swollen. The mucous membrane and perichondrium were divided close to the upper border of the cartilage and turned off, the superior cornu being divided and left. The outer surface was thus easily freed. Then the inferior cornu was cut off, and here some little difficulty was next experienced in dividing the ligament and muscles. The mucous membrane was next divided below the cords and back to the arytenoid, and the ala being drawn forward the attachment of the arytenoid to the cricoid was severed and this cartilage dissected out of its mucous membrane covering. Then the anterior two-thirds of the right cord were removed, the end stitched forward with catgut. One vessel, a branch of the superior thyroid, required a catgut ligature. Hahn's tube did not completely prevent blood trickling into the trachea, but a sponge passed into the cricoid ring above the tube effectually accomplished this, so that no trouble of any kind occurred during the operation. Next day Hahn's tube was replaced by an ordinary cannula, surrounded with sufficient gauze to plug the trachea. He was fed for five days with an œsophageal tube. On the sixth he was able to swallow. On the seventh he got up, and left, three weeks after the operation, quite well. His voice was clear and good, and was said by his friends to be better than before. Recurrence took place rapidly, and in December he had to be tracheotomised. Later the growth was seen budding upwards on the right side, but being still purely intra-laryngeal, total extirpation was proposed, and was carried out on December 22d, seven weeks from the first operation. Hahn's tube was again used, and the median cicatrix opened. The larynx was isolated by dividing the attached muscles on both sides, and it was noticed that the left side had become quite hard. Then the cricoid was separated from the trachea and raised from the epiglottis without injury. The connection of the pharynx to the back of the thyroid was divided, and the epiglottis cut through in its lower third. The immense cavity was stuffed as before, and a feeding tube passed by the mouth and retained. This tube was removed in two days, and was passed every four hours by the wound. In ten days he was up and about. On the eighth he swallowed, and by the end of January he had improved in health, and was quite well, save for the escape of saliva through the wound above the cannula. No complication of any kind occurred. He had no pulmonary complication at any time. Mr. Symonds found that the best method of dressing was to use a square of gauze, and pass its center well down into the cricoid, and then to pack this veil full of strips. The upper part of the wound was not packed so tightly as the lower part, and this allowed the saliva to escape. The patient found also that fluids were best swallowed after the second operation by lying flat upon his back. The patient was shown to the Fellows of the Society. He was in good health and spirits, and there was no sign of recurrence after four months. He could speak in a low, distinct, though gruff voice, and this was managed by passing through the upper wall of the cannula a curved tube which ran up to the base of the epiglottis, and admitted the air when he closed the cannula with the finger. This was found much simpler than the artificial larynx, and, moreover, the patient preferred the sound of his own voice. The vibrating structure appeared to be the mucous membrane of the pharynx running back from the epiglottis. This the authors thought was the first case on record in which the patient was able to speak after total extirpation of the larynx. Mr. Symonds pointed out that he was encouraged to obtain this result on account of a similar case he had heard of through a friend in Berlin. The parts removed were also exhibited. The primary growth was as big as a small hazel nut, while the recurrent one reached from the epiglottis to the lower margin of the cricoid, and had grown forward into the ala of the thyroid. It appeared to have originated in the stump of the right cord, which had been left. A microscopic examination of the growth confirmed the epitheliomatous characters. The artificial larynx, which had been obtained from Vienna, was also shown. The reed emitted a sound like an inferior toy trumpet, and could not be compared in efficiency to the voice possessed by the patient. The patient was able to speak in a loud though gruff voice simply by sending the air upwards through an aperture in the cannula. This was provided for by a large Durham's cannula, with an opening on the upper wall immediately behind the shield, through which passed a short curved tube in a direction upwards and then slightly forwards, projecting above the lower tube about three-fourths of an inch. In the posterior wall of the upper tube was an opening to admit air to and from the tracheal tube; the pharyngeal mucous membrane, falling in folds over the upper opening, was thrown into vibration, and a good vibrating voice was produced. During deglutition the upper tube was replaced by another of the same size and shape, but closed above so as to prevent the passage of food, etc., into the tracheal cannula. The present laryngoscopic appearances were as follows:—The epiglottis appeared perfectly normal; behind this one saw a funnel bounded by the lateral and posterior pharyngeal walls and the epiglottis; the apex of this funnel was composed of two folds of mucous membrane running antero-posteriorly, from between which, anteriorly, the air passed during phonation, causing them to vibrate. It was now more than four months since the second operation, there was no sign of recurrence, and the man was in good health.

